# Maresin 1 Ameliorates Diabetic Kidney Disease in Mice by Promoting Macrophage M2 Polarization

**DOI:** 10.1155/mi/4334095

**Published:** 2026-03-26

**Authors:** Yueli Pu, Xiumei Ma, Kang Geng, Renliang Meng, Yonglin Li, Chunmei Zheng, Changying Zhao, Fangyuan Teng, Yong Xu

**Affiliations:** ^1^ Department of Endocrinology and Metabolism, The Affiliated Hospital of Southwest Medical University, Luzhou, 646000, Sichuan, China, ahswmu.cn; ^2^ Metabolic Vascular Diseases Key Laboratory of Sichuan Province, Sichuan-Chongqing Joint Key Laboratory of Metabolic Vascular Diseases, Luzhou, 646000, Sichuan, China; ^3^ Sichuan Clinical Research Center for Nephropathy, Sichuan Clinical Research Center for Diabetes and Metabolic Disease, Luzhou, 646000, Sichuan, China; ^4^ Department of Endocrinology and Metabolism, The Affiliated Traditional Chinese Medical Hospital of Southwest Medical University, Luzhou, 646000, Sichuan, China, swmu.edu.cn; ^5^ Sichuan Provincial Key Laboratory for Human Disease Gene Study and Department of Laboratory Medicine, Sichuan Provincial People’s Hospital, University of Electronic Science and Technology of China, Chengdu, 610000, Sichuan, China, uestc.edu.cn; ^6^ Department of Plastic and Burns Surgery, National Key Clinical Construction Specialty, The Affiliated Hospital of Southwest Medical University, Luzhou, 646000, Sichuan, China, ahswmu.cn; ^7^ Department of Neurology, The Affiliated Hospital of Southwest Medical University, Luzhou, 646000, Sichuan, China, ahswmu.cn

**Keywords:** diabetic kidney disease, immune modulation, macrophage polarization, Maresin 1

## Abstract

**Background:**

The progression of diabetic kidney disease (DKD) is strongly associated with a chronic inflammatory microenvironment, with macrophage polarization imbalance recognized as a pivotal driver. Maresin 1 (MaR1), a specialized proresolving lipid mediator, plays a crucial role in restoring immune homeostasis across various inflammatory conditions. However, its precise role in ameliorating kidney injury through macrophage polarization in DKD remains unclear.

**Methods:**

The type 2 diabetes mouse model was established using a high‐fat diet combined with streptozotocin induction. The therapeutic efficacy of MaR1 was assessed by evaluating metabolic parameters (blood glucose, lipid profile), renal function (urine albumin‐to‐creatinine ratio [ACR], serum creatinine, and blood urea nitrogen [BUN]), and renal pathology (HE staining and Masson staining). In vitro, bone marrow‐derived macrophages (BMDMs) were exposed to high glucose, and the modulatory effect of MaR1 on M1/M2 polarization was assessed using RT‐qPCR, immunohistochemistry, and immunofluorescence.

**Results:**

MaR1 treatment significantly ameliorated metabolic abnormalities in diabetic mice (lower blood glucose and cholesterol), improved renal function (reduced ACR, serum creatinine, and BUN), and attenuated renal fibrosis (all *p*  < 0.05). Mechanistically, MaR1 reversed macrophage polarization imbalance both in vivo and in vitro, promoting a shift from the M1 phenotype (downregulation of iNOS and TNF‐α) to the M2 phenotype (upregulation of Arg‐1 and IL‐10).

**Conclusion:**

MaR1 improves metabolic disturbances and renal injury in DKD by driving macrophage polarization toward the M2 phenotype and restoring immune homeostasis. These findings highlight MaR1 as a promising candidate for targeted immunomodulatory therapies in DKD.

## 1. Introduction

Diabetic kidney disease (DKD), one of the most serious microvascular complications of diabetes, affects ~40% of diabetes patients worldwide and is the leading cause of end‐stage renal disease (ESRD) [1, 2]. The hallmark pathological features of DKD—glomerulosclerosis, mesangial matrix expansion, and progressive interstitial fibrosis—are driven by a vicious cycle of hyperglycemia, lipid dysregulation, and chronic inflammation [3]. Although current therapies—such as glycemic control, antihypertensive agents, and renin–angiotensin system inhibitors—can delay disease progression [4], many patients inevitably develop ESRD, underscoring the urgent need for novel therapeutic strategies targeting the inflammatory microenvironment.

Specialized proresolving mediators (SPMs) are endogenous lipid molecules with dual actions of inflammation resolution and tissue repair [5, 6]. Among them, Maresin 1 (MaR1) has demonstrated potent anti‐inflammatory and tissue‐protective effects in metabolic diseases such as atherosclerosis and obesity‐associated inflammation [7–10]. However, its regulatory role within the renal microenvironment of DKD remains poorly understood.

Macrophage polarization is a central determinant of DKD pathogenesis. Classically activated M1 macrophages aggravate tissue damage through the release of proinflammatory cytokines such as IL‐6 and TNF‐α, whereas alternatively activated M2 macrophages mitigate injury and promote repair by producing anti‐inflammatory mediators such as IL‐10 [11, 12]. An imbalance favoring the M1 phenotype perpetuates renal injury and fibrosis [13]. Thus, therapeutic interventions that promote macrophage reprograming toward the M2 phenotype have emerged as a promising strategy to slow DKD progression. Yet, whether exogenous SPMs such as MaR1 can modulate this process through defined intracellular signaling pathways remains largely unexplored.

In this study, we systematically investigated the effects of MaR1 in a mouse model of DKD and in primary bone marrow‐derived macrophages (BMDMs) exposed to high glucose (HG) conditions. We aimed to elucidate (i) the ability of MaR1 to improve metabolic parameters, renal function and fibrosis and (ii) its effects on macrophage M1/M2 polarization. These findings may provide novel insights for the development of immunomodulatory strategies in DKD.

## 2. Materials and Methods

### 2.1. Animal Experiment

A total of 19 male C57BL/6J mice aged 6 weeks (Nanjing Jicui Pharmaceutical Biotechnology Co., Ltd, China) were used in this study. The mice were randomly divided into the following experimental groups: normal control mice (NC group, *n* = 6), high‐fat diet (HFD, 60% fat) combined with continuous intraperitoneal injection of low‐dose streptozotocin (STZ, Beijing Solarbio Technology Co., Ltd, China)‐induced T2DM mice (DKD group, *n* = 7), and T2DM mice treated with MaR1 (DKD + MaR1 group, *n* = 6). We induced the T2DM model with a high‐fat diet. Mice received an intraperitoneal injection of STZ at a dose of 50 mg/kg for 7 consecutive days. Mice with fasting blood glucose levels exceeding 16.7 mM were identified as diabetic [14]. After STZ injection, MaR1 (Cayman, USA) was administered at a dose of 4 μg/kg via intraperitoneal injection once every 24 h for 12 weeks [15]. The mice were housed in individually ventilated cages at a temperature of 23 ± 1°C, with a 12‐h light–dark cycle and relative humidity of 60 ± 10%. All mice were euthanized after 12 weeks of treatment. During the experiment, body weight was recorded weekly, and blood glucose was recorded every 2 weeks. The animal experiment was approved by the Animal Ethics Committee of Southwest Medical University (Number 20220225–014) and complied with the National Institutes of Health (NIH) guidelines for the care and use of laboratory animals. Before the mice were euthanized, urine samples were collected by using the Single Mouse Metabolic Cage (Tecniplast, Italy).

### 2.2. Mouse Primary Macrophage Culture

To isolate primary BMDMs from the bone marrow of male C57BL/6J mice, the tibia was taken, sterilized with 75% alcohol, and rinsed with precooled culture medium. The ends of the tibia were cut off, and the bone marrow cells were flushed out with culture medium, yielding red bone marrow. The bone marrow was slowly blown to obtain a single‐cell suspension. The cell suspension was centrifuged at 4°C, 1600 rpm for 5 min, and the supernatant was discarded. Red blood cells were lysed using a red blood cell lysis solution for no more than 1 min. Subsequently, the red blood cells were washed twice with precooled RPMI 1640 culture medium at 4°C, 1600 rpm. The cells were then resuspended in RPMI 1640 culture medium containing 10% heat‐inactivated fetal bovine serum (FBS) and 20 ng/mL recombinant mouse macrophage colony‐stimulating factor (M‐CSF) at a density of 5 × 10^5^ cells/well in a 6‐well plate and placed in an incubator. After 3 days, the culture medium and nonadherent cells were removed, and the adherent macrophages were further cultured for 3 days for different treatments. The MTT method was used to screen for the optimal concentration of MaR1 (10 nM), and mannitol at an equimolar concentration was regarded as an osmotic control to eliminate the influence of hypertonic environments on macrophage polarization. BMDMs were divided into three groups: low glucose (LG, 5.6 mM D‐glucose), high glucose (HG, 30 mM D‐glucose), and high glucose plus MaR1 (HG + MaR1, 30 mM D‐glucose with 10 nM MaR1). Treatments lasted for 72 h.

### 2.3. Assessment of Lipids, Renal Function, Inflammatory Cytokines, and Anti‐Inflammatory Factor

Serum total cholesterol (TC) levels were measured using an Olympus AU400 automated chemistry analyzer (Olympus, Japan) following the manufacturer’s protocol. Commercially available assay kits (Nanjing Jiancheng Bioengineering Institute, China) were used to determine blood urea nitrogen (BUN) and serum creatinine in mice. Commercial ELISA kits (Andygene, USA) were used to measure TNF‐α, IL‐6, and IL‐10 levels in mice serum and BMDM cell culture supernatants, as well as albumin levels in mice urine. All assays were performed according to the kit instructions.

### 2.4. HE Staining and Masson Staining

Mice kidney tissue was fixed with 4% paraformaldehyde (PFA), paraffin‐embedded, and sectioned into 4 μm thick slices for histological analysis. Kidney sections were stained, dewaxed, and mounted according to the instructions of commercial HE (hematoxylin‐eosin) staining kits and Masson staining kits (Beijing Solarbio Technology Co., Ltd, China). Finally, renal tissue architecture and fibrosis were examined under a microscope.

### 2.5. Immunofluorescence

Cells were permeabilized with 2% Triton X‐100 for 15 min, followed by treatment with 2 M hydrochloric acid for 20 min. Cells were blocked with 2% bovine serum albumin (BSA) for 45 min and then incubated overnight at 4°C with primary antibodies against F4/80 (co‐expression in macrophages), iNOS (M1 macrophages), or Arg‐1 (M2 macrophages). Subsequently, cells were incubated at room temperature for 2 h with goat antirabbit IgG H&L fluorescent secondary antibody or goat antimouse IgG H&L fluorescent secondary antibody, followed by DAPI (2 μg/mL) staining. The cells were observed under an Olympus BX51 fluorescence microscope after mounting.

### 2.6. Immunohistochemistry

About 4 μm kidney sections were deparaffinized and hydrated, then stained with anti‐iNOS (1:100, Cell Signaling Technology, USA) or anti‐Arg‐1 (1: 100, Cell Signaling Technology, USA) primary antibody. The sections were then stained with biotin‐labeled goat antirabbit IgG or biotin‐labeled antimouse IgG, followed by treatment with streptavidin‐horseradish peroxidase conjugate. Photographs of each stained section were taken using an optical microscope.

### 2.7. RT‐qPCR

Total RNA from kidney tissues and BMDMs was extracted using Trizol reagent (Invitrogen, USA). Reverse transcription reactions were performed using ReverTra Ace qPCR reverse transcription premix (TOYOBO, China), and real‐time quantitative PCR (RT‐qPCR) was conducted using the QuantiNova SYBR Green PCR kit (QIAGEN, Germany). Real‐time qPCR was performed on the Analytik Jena qTOWER 3G real‐time PCR system (JENA, Germany) following the manufacturer’s instructions. The primers used in this study are listed in Table 1. GAPDH was selected as the internal control. All samples were set with three replicates. The relative expression of target genes was calculated using the 2^−△△Ct^ method relative to the reference gene.

**Table 1:** Primer sequences for RT‐qPCR analysis.

Gene name	Sequence (5′ to 3′)	
GAPDH	Forward	ACTCCACTCACGGCAAATTCA
	Reverse	CGCTCCTGGAAGATGGTG
Arg‐1	Forward	CATGGGCAACCTGTGTCCTT
	Reverse	CGATGTCTTTGGCAGATATGCA
iNOS	Forward	GCCCTGCTTTGTGCGAAGTG
	Reverse	AGCCCTTTGTGCTGGGAGTC
IL‐10	Forward	TTTGAATTCCCTGGGTGAGAA
	Reverse	CTCCACTGCCTTGCTCTTATTTTC
TNF‐α	Forward	CATCTTCTCAAAATTCGAGTGACAA
	Reverse	TGGGAGTAGACAAGGTACAACCC

### 2.8. Statistical Analyses

All data were analyzed using SPSS 27.0 software (IBM, Armonk, NY, USA). Measurement data were expressed as mean ± SD (standard deviation). For two independent groups with normal distribution and equal variance, independent sample *t*‐tests were used for comparison; for multiple groups, one‐way ANOVA combined with Tukey’s post hoc test was used. A *p*‐value of < 0.05 was considered statistically significant.

## 3. Results

### 3.1. MaR1 Improves Metabolic and Renal Parameters in Diabetic Mice

Following 1 week of adaptive feeding, the mice were randomly assigned to a normal control group (*n* = 6) and an HFD group (*n* = 13). At the 11th week, mice in the HFD group received an intraperitoneal injection of streptozotocin to induce a T2DM model. Upon successful model establishment, they were further randomly subdivided into the DKD group (*n* = 7) and DKD + MaR1 group (*n* = 6). Starting from the 12th week, and the DKD + MaR1 group received intraperitoneal injections of MaR1. All mice in the DKD and DKD + MaR1 groups continued to be maintained on the high‐fat diet (Figure 1a).

Figure 1MaR1 ameliorates metabolic abnormalities, renal function, and renal fibrosis in DKD mice. (a) Experimental design for MaR1 treatment of HFD‐STZ‐induced DKD mice; (b) changes in body weight among experimental groups; (c) blood glucose levels; (d) serum total cholesterol concentrations; (e) serum creatinine levels; (f) blood urea nitrogen (BUN); (g) representative HE staining and Masson staining of kidney sections showing renal fibrosis, bar: 80 um; (h) urinary albumin‐to‐creatinine ratio (ACR). Data are shown as mean ± SD.  ^∗^ *p* < 0.01;  ^∗∗^ *p* < 0.01;  ^∗∗∗^ *p* < 0.0001,  ^∗∗∗∗^ *p* < 0.0001. NC: normal control; DKD: diabetic kidney disease; DKD + MaR1: DKD mice treated with MaR1.

**Figure:**
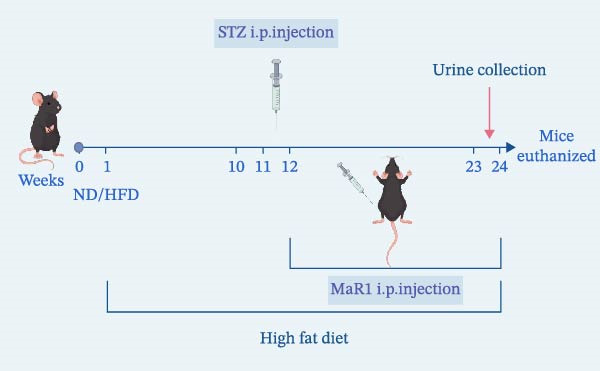
(a)

**Figure:**
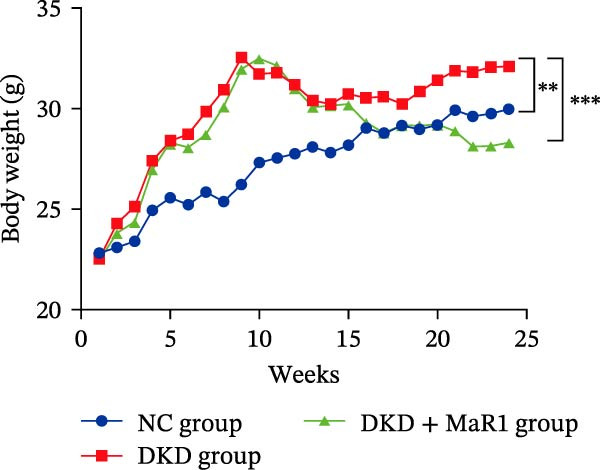
(b)

**Figure:**
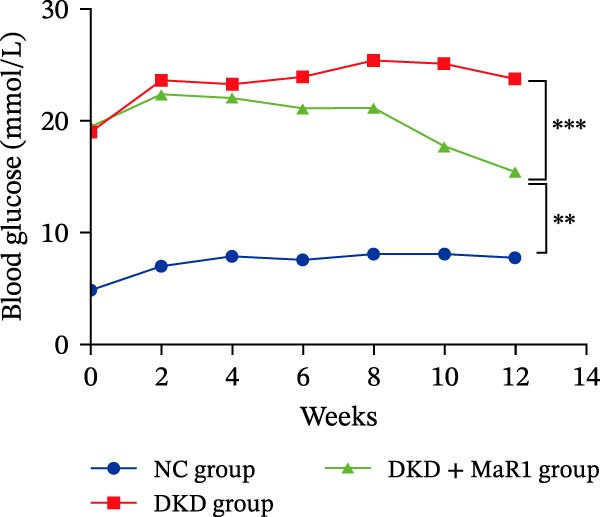
(c)

**Figure:**
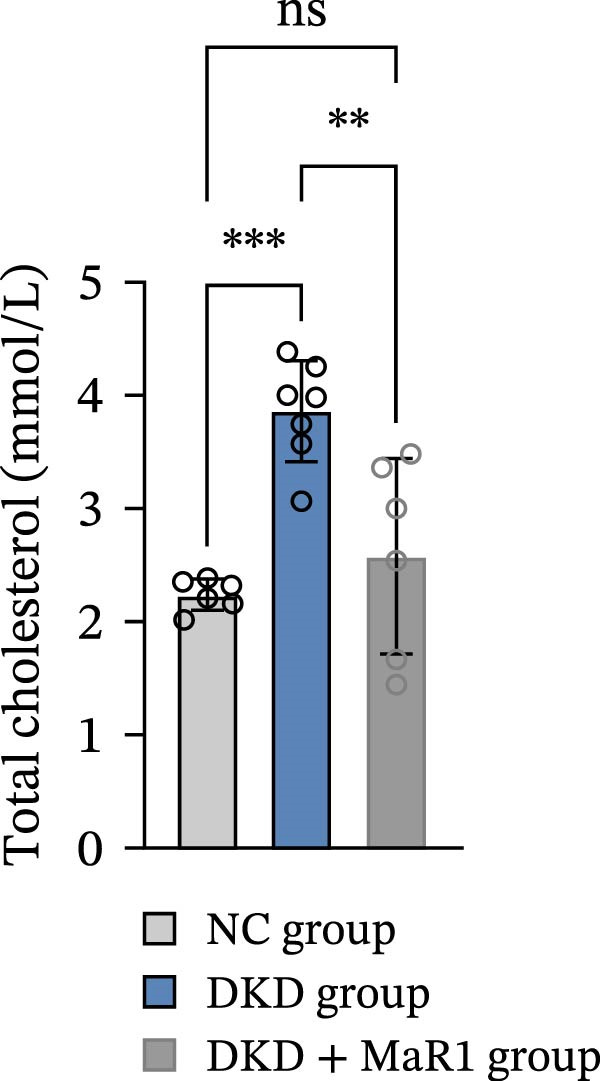
(d)

**Figure:**
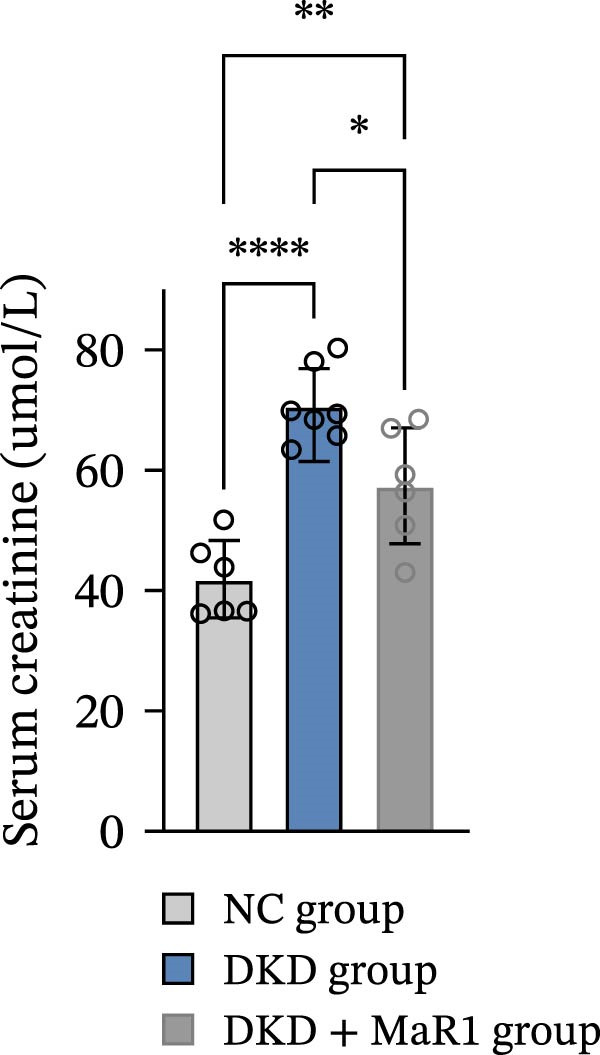
(e)

**Figure:**
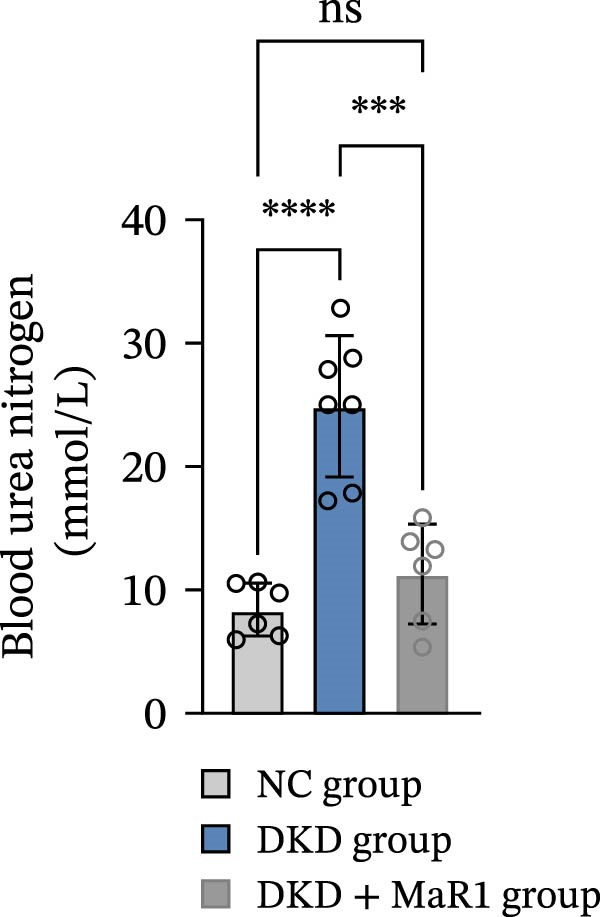
(f)

**Figure:**
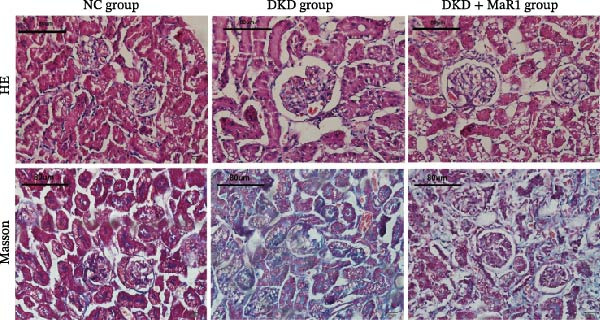
(g)

**Figure:**
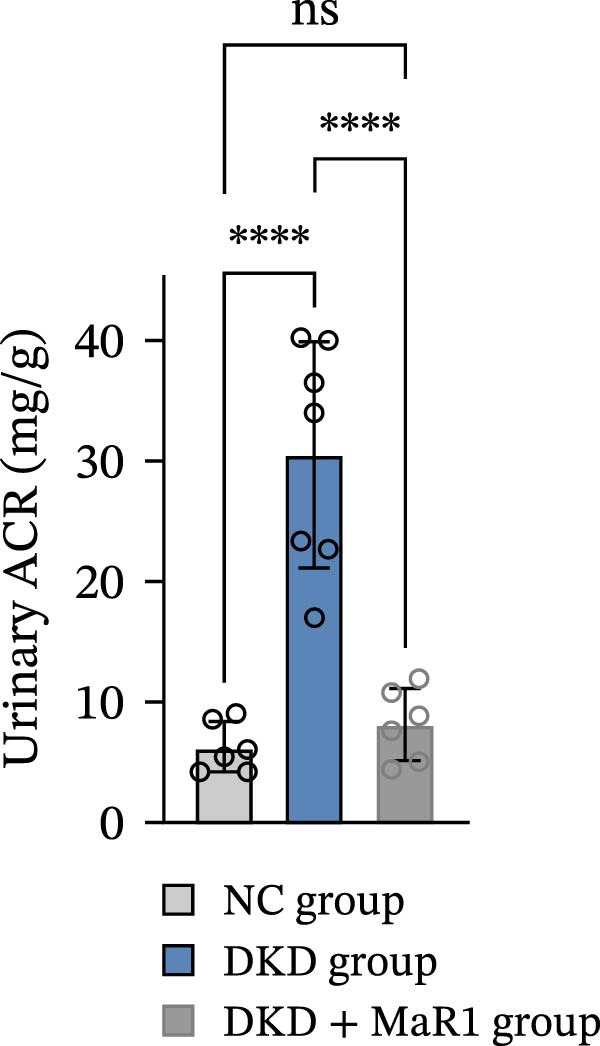
(h)

Metabolic analysis revealed significant difference in body weight between the NC group and DKD group (*p* < 0.05). Meanwhile, the DKD + MaR1 group significantly reduced body weight compared to the DKD group (*p* < 0.05; Figure 1b). In terms of blood glucose regulation, the blood glucose in the DKD group was significantly higher than that in the NC group (*p* < 0.05), but MaR1 intervention significantly reduced blood glucose levels (vs. the DKD group, *p*  < 0.05) (Figure 1c). Blood lipid tests further confirmed that serum TC levels in the DKD group were significantly higher than those in the NC group (*p* < 0.05), while MaR1 intervention effectively reversed lipid abnormalities (TC significantly decreased vs. the DKD group, *p*  < 0.05) (Figure 1d), showing that the combination of a high‐fat diet and STZ effectively created the DKD model and that MaR1 improved metabolic disorders.

Regarding renal function and pathological changes: serum creatinine, BUN, and urinary albumin‐to‐creatinine ratio (ACR) in the DKD group were significantly higher than those in the NC group (*p* < 0.05), while MaR1 intervention significantly reduced serum creatinine, BUN, and urinary ACR levels (vs. the DKD group, *p*  < 0.05) (Figure 1e, f, h). Histological examination of kidney tissues (HE staining and Masson staining) showed that the DKD group exhibited typical pathological changes of DKD, including glomerular sclerosis, mesangial matrix hyperplasia, and extensive renal interstitial fibrosis, while MaR1 intervention significantly alleviated the degree of fibrosis (Figure 1g). Collectively, these results demonstrate that MaR1 reduces proteinuria, improves renal function, and alleviates fibrosis, thereby effectively attenuating the progression of DKD.

### 3.2. MaR1 Promotes M2 Macrophage Polarization in Renal Tissues

Immunohistochemical analysis showed that the expression of the M1 macrophage marker iNOS in the kidney tissues of the DKD group was significantly upregulated compared to the NC group (*p* < 0.05), while the expression of the M2 marker Arg‐1 showed no statistical difference (Figure 2a). After MaR1 intervention, the mRNA expression of iNOS in the DKD + MaR1 group was significantly lower than that in the DKD group (*p* < 0.05), while the mRNA expression of Arg‐1 significantly increased (*p* < 0.05), indicating a shift in macrophage phenotype from M1 dominance to M2 (Figure 2b, c).

Figure 2MaR1 promotes M2 macrophage polarization in DKD kidneys. (a) Immunohistochemical staining of renal tissues for iNOS (M1 marker) and Arg‐1 (M2 marker), bar: 80 um; (b–c) RT‐qPCR analysis of iNOS and Arg‐1 mRNA levels in mice kidney tissues.  ^∗^ *p* < 0.05;  ^∗∗^ *p*   < 0.01;  ^∗∗∗^ *p*   < 0.001,  ^∗∗∗∗^ *p* < 0.0001.

**Figure:**
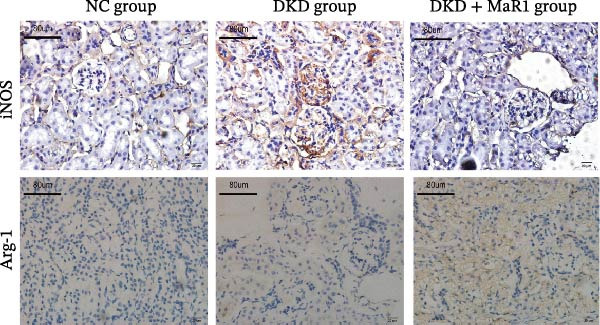
(a)

**Figure:**
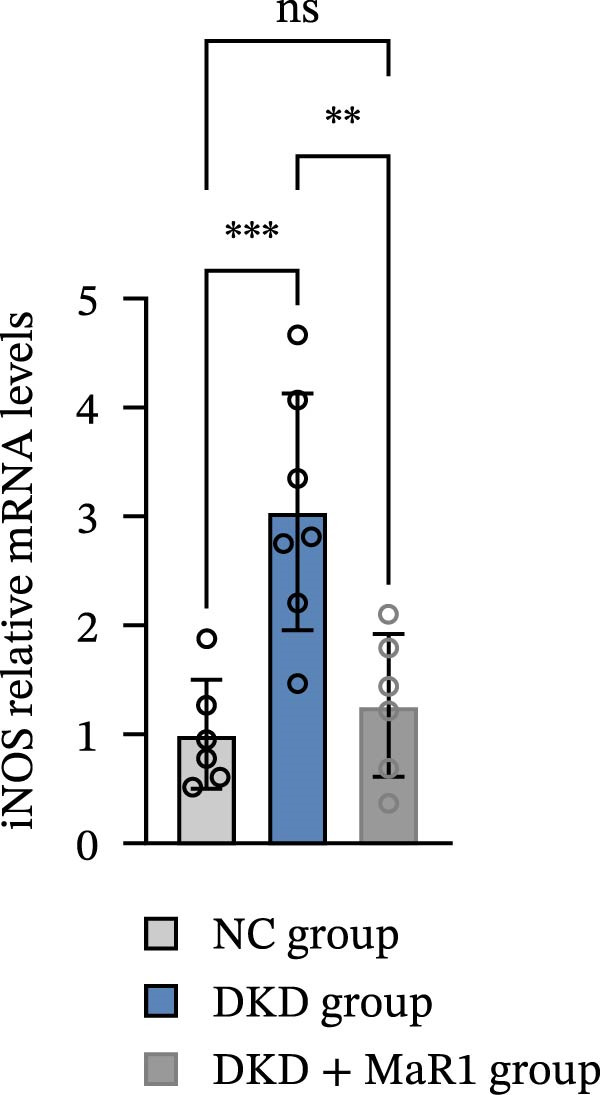
(b)

**Figure:**
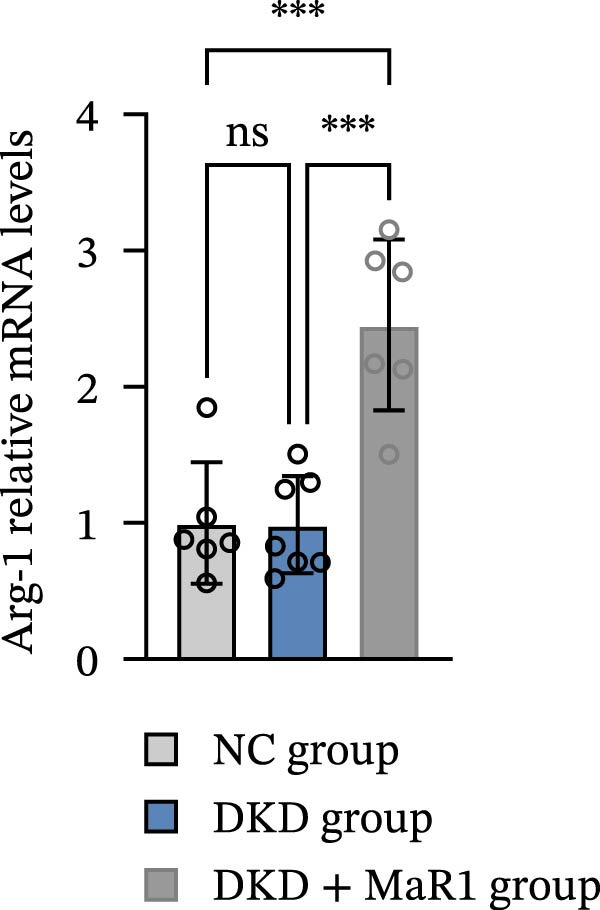
(c)

Serum inflammatory factor detection further validated this phenotype conversion: the levels of proinflammatory factors IL‐6 and TNF‐α in the DKD group were significantly higher than those in the NC group (*p* < 0.05), while MaR1 intervention significantly reduced IL‐6 and TNF‐α levels (vs. the DKD group, *p*  < 0.05) and significantly increased the level of the anti‐inflammatory factor IL‐10 (vs. the NC and DKD groups, both *p*  < 0.05) (Figure 3a, b, c). Results at the gene and protein expression levels were consistent: RT‐qPCR showed that the mRNA levels of TNF‐α in the kidneys of the DKD group were significantly higher than those in the NC group (*p* < 0.05), and were significantly downregulated after MaR1 intervention (*p* < 0.05); meanwhile, the mRNA levels of IL‐10 were significantly upregulated in the MaR1 intervention group compared to both the NC and DKD groups (*p* < 0.05)(Figure 3d, e).

Figure 3MaR1 reduces proinflammatory cytokines and enhances anti‐inflammatory factor expression in DKD mice. (a) Serum IL‐6 concentrations; (b) serum TNF‐α concentrations; (c) serum IL‐10 concentrations; (d–e) RT‐qPCR analysis of TNF‐α and IL‐10 mRNA levels in mice kidney tissues. 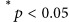; 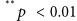; 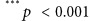, 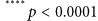.

**Figure:**
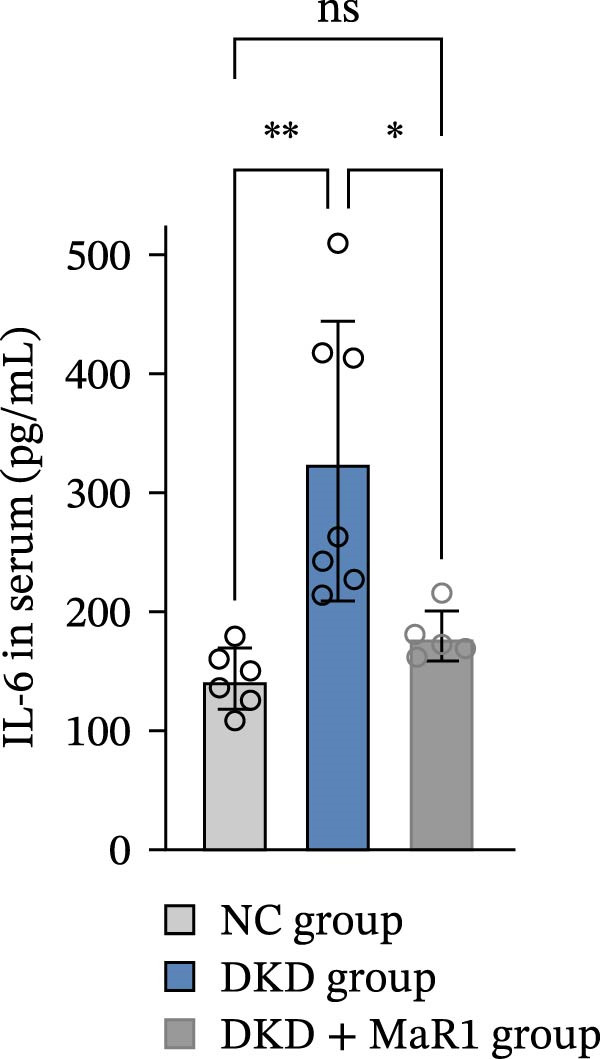
(a)

**Figure:**
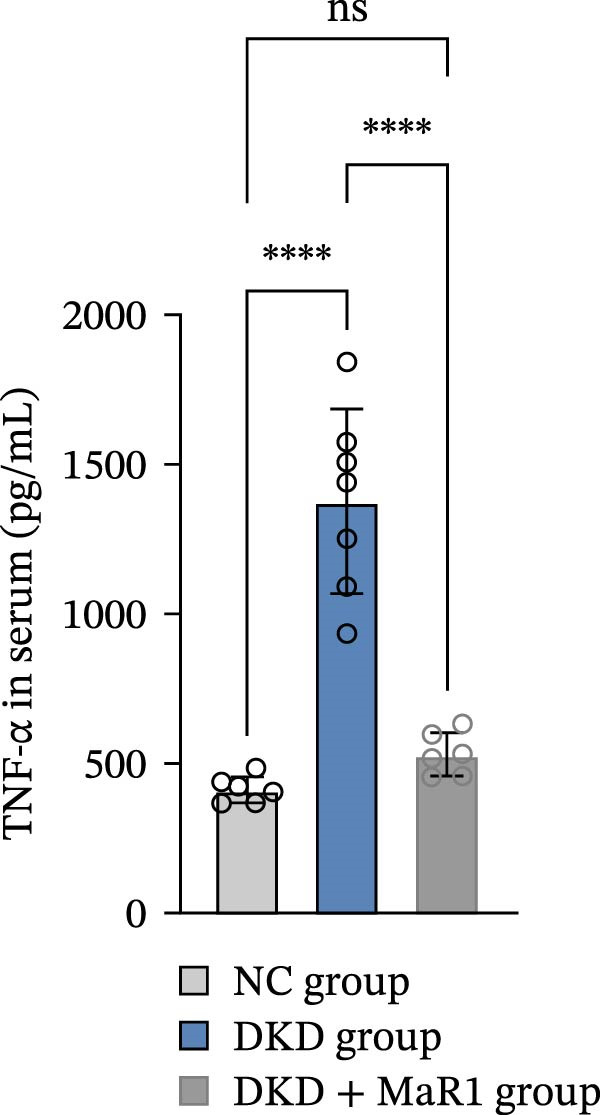
(b)

**Figure:**
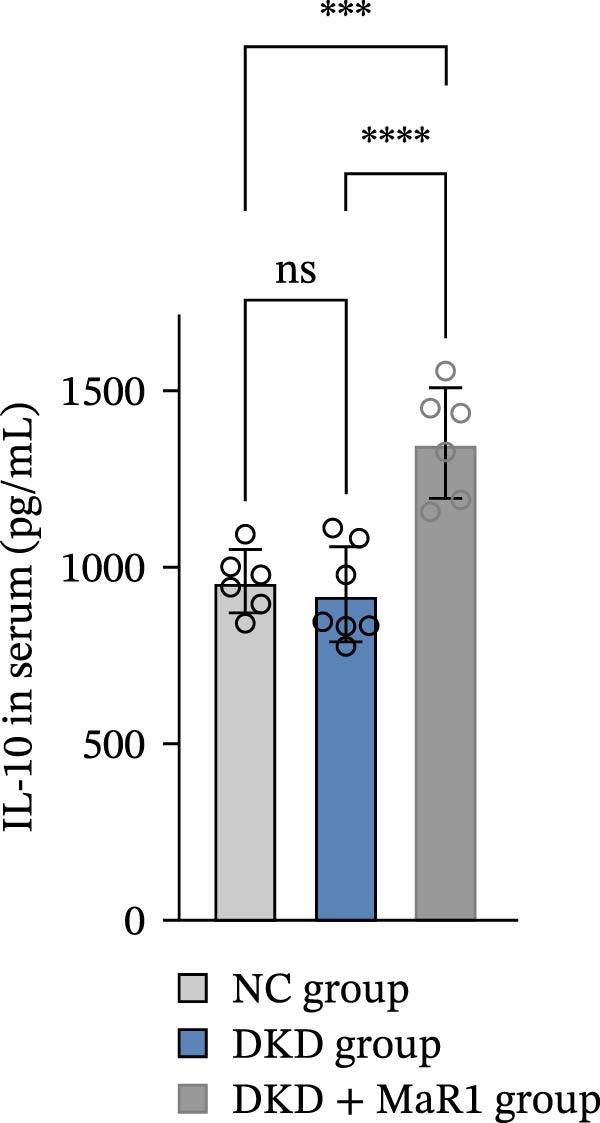
(c)

**Figure:**
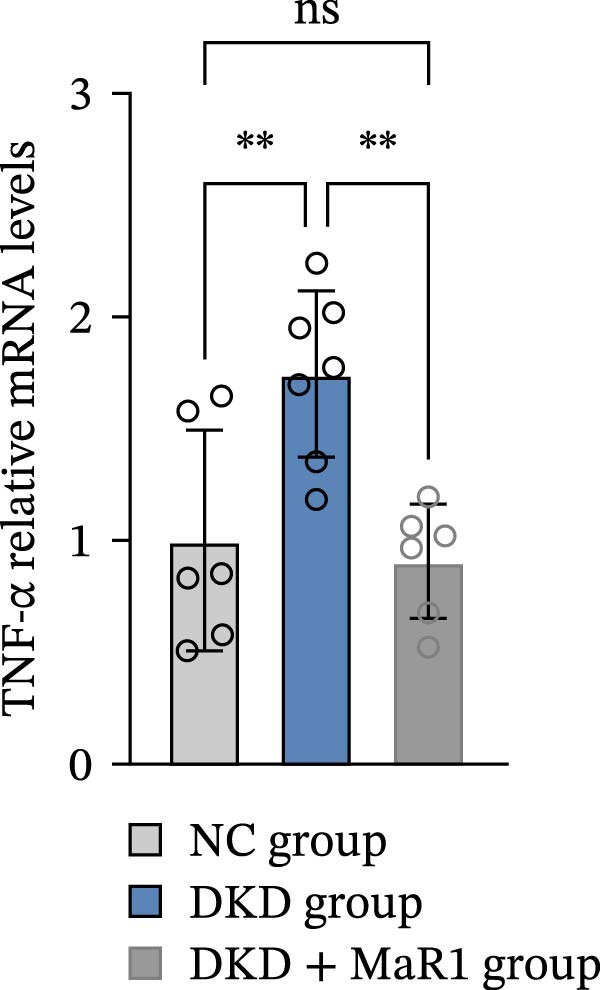
(d)

**Figure:**
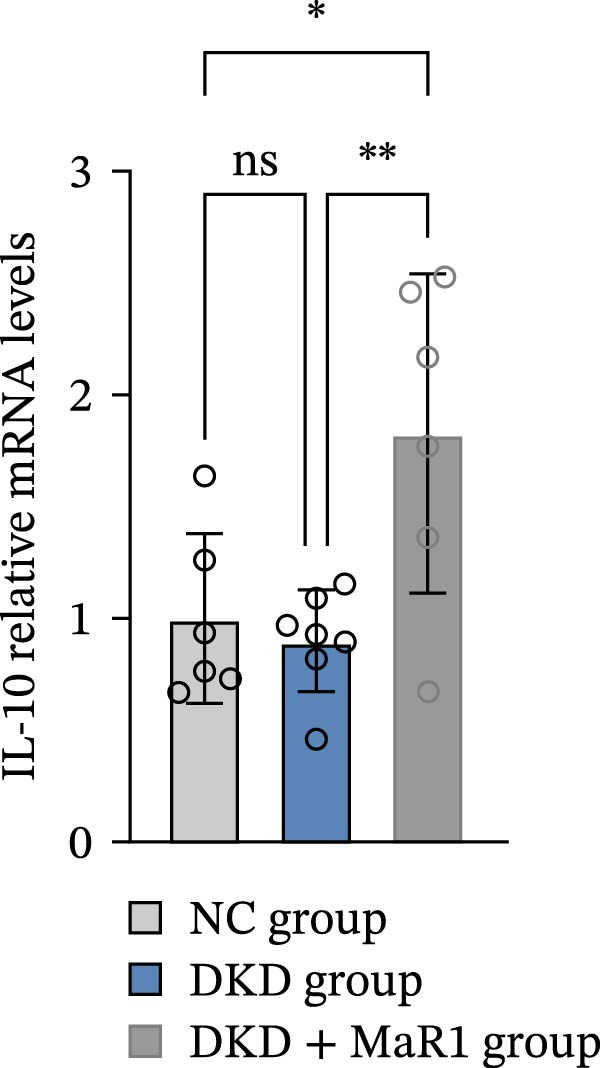
(e)

In summary, the renal microenvironment in DKD is characterized by M1 macrophage infiltration, and MaR1 intervention promotes macrophage polarization towards the M2 macrophage, coordinately reducing the release of proinflammatory factors (IL‐6 and TNF‐α) and increasing the secretion of anti‐inflammatory factors (IL‐10), thereby exerting anti‐inflammatory and inflammation‐resolving effects.

### 3.3. MaR1 Reverses HG‐Induced M1 Polarization in BMDMs

In the in vitro BMDM model, we compared the effects of LG, HG, and high glucose + MaR1 (HG + MaR1) treatments. Cytokine detection showed that compared to the LG group, HG stimulation significantly upregulated the level of the proinflammatory factor TNF‐α in the macrophage supernatant (*p* < 0.05) (Figure 4a); however, MaR1 intervention (HG + MaR1 group) significantly reduced TNF‐α expression compared to the HG group (*p* < 0.05) (Figure 4a), while increasing the level of the anti‐inflammatory factor IL‐10 (*p* < 0.05) (Figure 4b).

Figure 4MaR1 reverses high glucose‐induced macrophage polarization and modulates cytokine secretion and gene expression. (a) Supernatant TNF‐α concentrations; (b) Supernatant IL‐10 concentrations; (c) Immunofluorescence staining of BMDMs showing iNOS and Arg‐1 expression, Bar: 100 um; (d–g) RT‐qPCR analysis of iNOS, Arg‐1, TNF‐α, and IL‐10 mRNA levels. LG: low glucose; HG: high glucose; HG + MaR1: high glucose with MaR1 treatment. 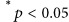; 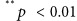; 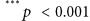, 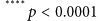.

**Figure:**
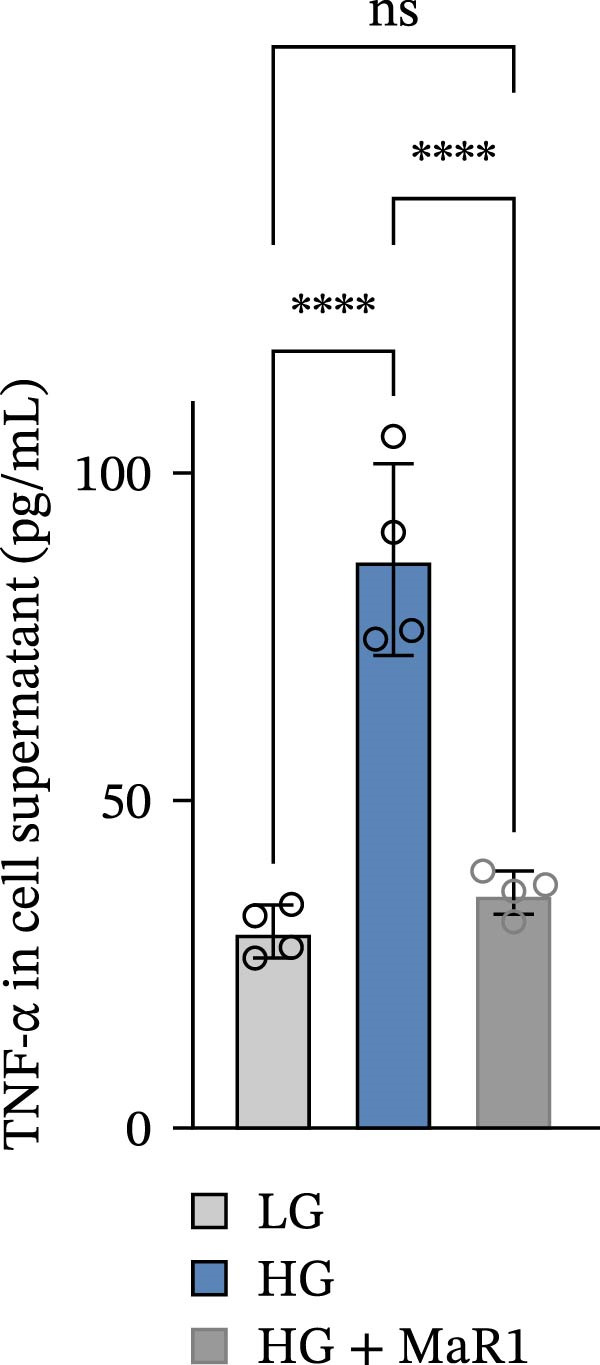
(a)

**Figure:**
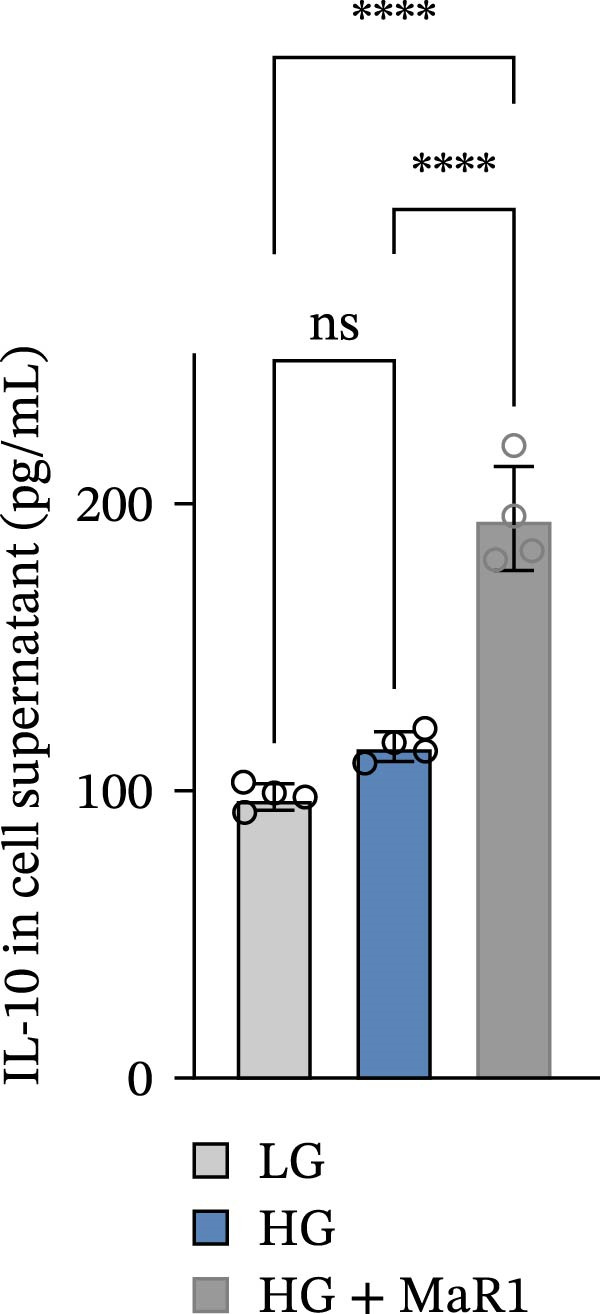
(b)

**Figure:**
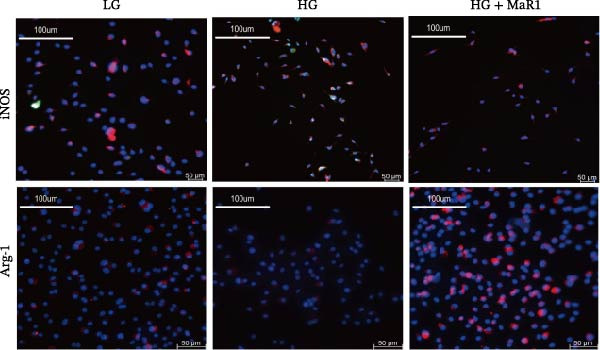
(c)

**Figure:**
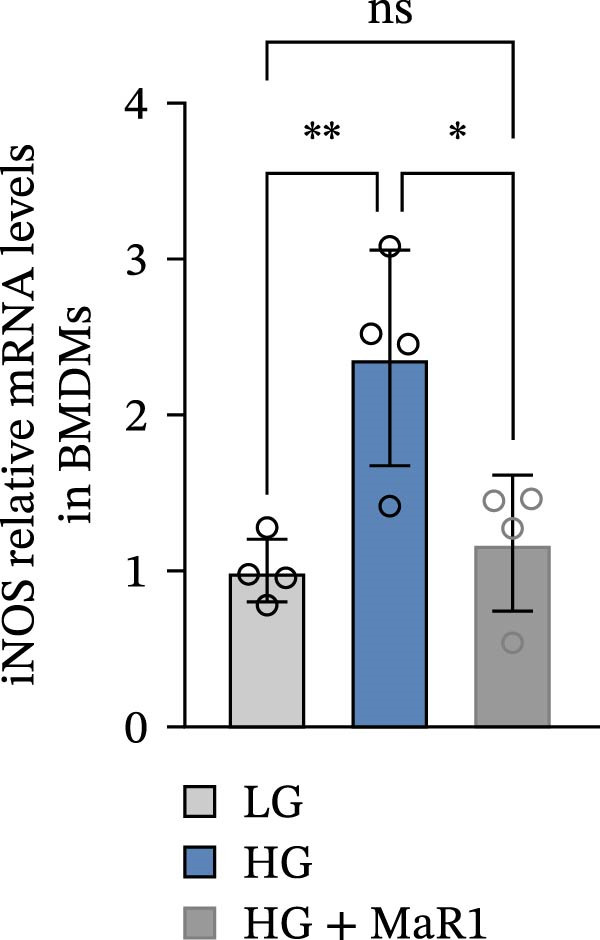
(d)

**Figure:**
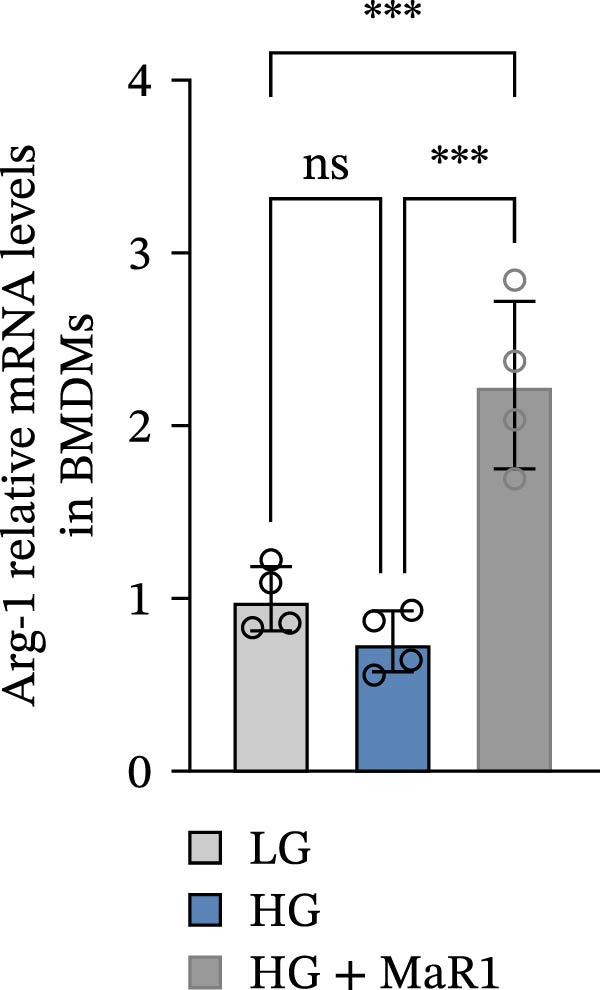
(e)

**Figure:**
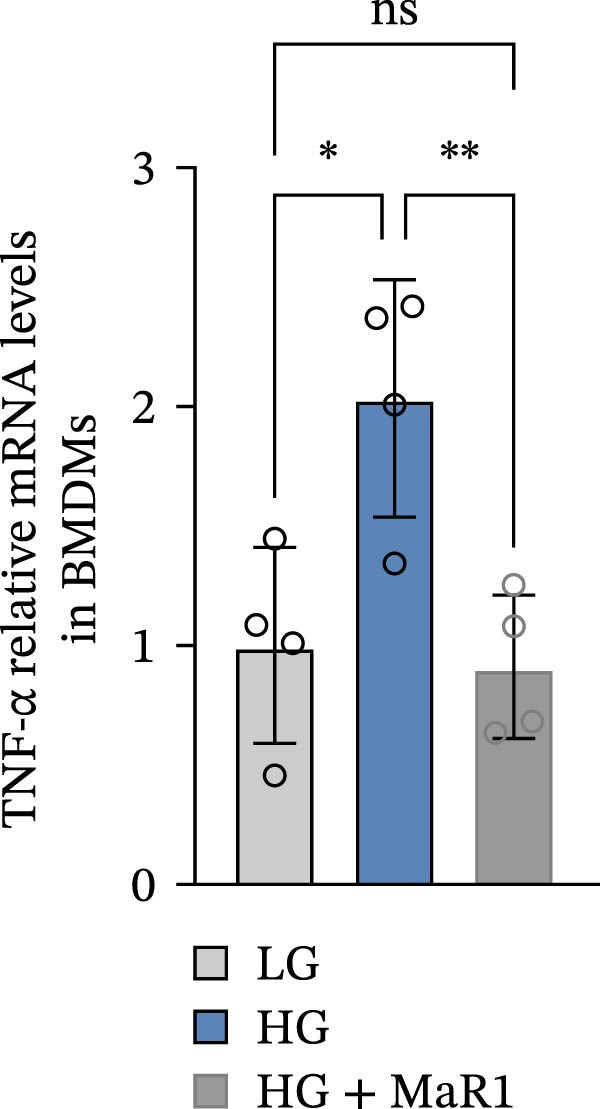
(f)

**Figure:**
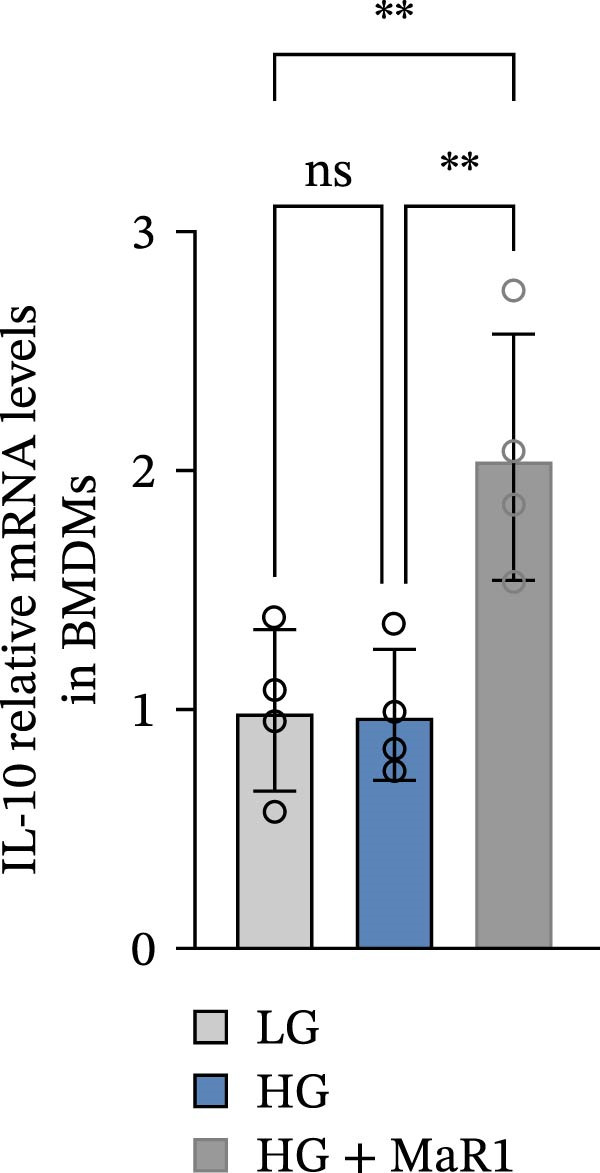
(g)

Immunofluorescence results further revealed phenotype changes: HG stimulation (HG group) significantly enhanced the expression of the M1 marker iNOS and suppressed the M2 marker Arg‐1 (vs. LG group), while MaR1 treatment completely reversed this effect (Figure 4c). Gene expression validation: the mRNA levels of proinflammatory phenotype‐related genes iNOS and TNF‐α in the HG group were significantly higher than those in the LG group (*p* < 0.05) (Figure 4d, f), and were significantly downregulated after MaR1 intervention (vs. HG group, *p*  < 0.05); MaR1 intervention significantly upregulated the mRNA levels of anti‐inflammatory phenotype‐related genes Arg‐1 and IL‐10 (vs. HG group, *p*  < 0.05)(Figure 4e, g).

## 4. Discussion

DKD has become a leading cause of ESRD worldwide and is characterized by persistent proteinuria, reduced glomerular filtration rate, and progressive renal fibrosis [16, 17]. Although intensive glycemic and blood pressure control can delay disease progression, existing therapeutic options remain insufficient to halt renal deterioration. Increasing evidence highlights chronic low‐grade inflammation, particularly macrophage‐driven immune dysregulation, as a central mechanism in DKD pathogenesis [17–19]. An imbalance favoring proinflammatory M1 macrophages over reparative M2 macrophages sustains a profibrotic renal microenvironment, thereby providing a rationale for macrophage‐targeted therapies [20, 21].

This study systematically explored the intervention effects of MaR1 on DKD and its mechanisms through in vivo and in vitro experiments, with the main findings as follows: MaR1 significantly improved metabolic disorders (body weight, blood glucose, blood lipids) and renal function damage in diabetic mice, with the main mechanism promoting renal macrophages to shift toward an anti‐inflammatory (M2) phenotype. The following is an in‐depth analysis of the results in conjunction with existing studies.

### 4.1. The Improvement Effect of MaR1 on Diabetes Metabolism and Renal Function and Its Pathological Mechanism

In this study, MaR1 intervention significantly reduced blood glucose and TC levels in diabetic mice, consistent with its metabolic regulatory roles in other conditions [22–24]. Notably, despite ongoing HFD feeding, MaR1‐treated mice exhibited reduced body weight. This likely reflects enhanced energy expenditure and improved metabolic homeostasis, consistent with the concurrent improvements in glycemia and lipid profiles. Prior studies indicate MaR1 activates brown adipose tissue and promotes white adipose tissue browning, increasing energy expenditure [8, 9]. The delayed weight difference may reflect the time required for adipose tissue remodeling.

Regarding the specific mechanism underlying the hypoglycemic effect, while direct evidence of MaR1 protecting pancreatic β‐cells in DKD is limited, its ability to improve systemic insulin sensitivity is well‐documented. MaR1 has been shown to enhance insulin signaling in peripheral tissues such as adipose tissue and muscle [8]. Therefore, the glucose‐lowering effect observed here is unlikely to be an incidental byproduct of its anti‐inflammatory action but rather an active component of its multifaceted metabolic regulation, which also includes the activation of brown adipose tissue and browning of white adipose tissue to increase energy expenditure [9]. Therefore, the modest weight reduction observed here is consistent with MaR1’s role in enhancing systemic energy expenditure, and it complements, rather than contradicts, the concurrent improvements in renal parameters. Thus, systemic metabolic improvement synergizes with local reprograming of renal macrophages toward the M2 phenotype, collectively alleviating metabolic toxicity and inflammation to confer comprehensive renal protection. Future studies directly examining pancreatic islet morphology and *in vivo* insulin sensitivity will be valuable to delineate the primary mechanism underlying the glucoregulatory effect of MaR1 in DKD.

Renal function indicators (serum creatinine, BUN, and ACR) and renal histological results (mesangial hyperplasia, alleviation of renal interstitial fibrosis) indicate that MaR1 can effectively alleviate the core pathological damage of DKD. Combining the combined effects of improving metabolism and regulating inflammation, it is speculated that MaR1 may exert protective effects through the “metabolism‐inflammation‐renal injury” axis: on one hand, it reduces metabolic toxicity’s direct damage to the kidneys by lowering blood glucose and lipids; on the other hand, it interrupts the vicious cycle of renal tissue damage by inhibiting inflammatory responses.

### 4.2. The Anti‐Inflammatory and Repair‐Promoting Effects of MaR1 in Regulating Macrophage Polarization

Macrophage phenotype imbalance (predominantly M1 type) is a core feature of chronic inflammation in DKD [25, 26]. In this study, the M1 marker protein iNOS (both mRNA and protein levels) and serum proinflammatory factors (IL‐6 and TNF‐α) in the DKD group were significantly upregulated, while the M2 marker protein Arg‐1 showed significant change, consistent with the pathological characteristics of DKD’s proinflammatory‐repair imbalance. After MaR1 intervention, iNOS expression was significantly downregulated, while Arg‐1 and the anti‐inflammatory factor IL‐10 were significantly upregulated, indicating that MaR1 can reshape macrophage phenotypes through a dual mechanism of inhibiting M1 activation and promoting M2 differentiation.

In vitro BMDM experiments further validated the direct action of MaR1: under HG stimulation, the levels of TNF‐α in the macrophage supernatant increased, and iNOS mRNA was upregulated, presenting typical M1 characteristics; after MaR1 intervention, TNF‐α decreased, IL‐10 increased, and Arg‐1 mRNA was upregulated, indicating that MaR1 can directly reverse HG‐induced M1 polarization. This result is similar to the action patterns of members of the SPMs family (such as Resolvin D1 [27, 28]), supporting MaR1’s core function as an “inflammation‐resolving mediator“ [29].

### 4.3. Potential Therapeutic Implications of MaR1 in Diabetic Multicomplications

Our findings highlight MaR1’s renoprotective effects. Importantly, diabetic complications often involve multiple organs sharing common inflammatory and metabolic pathways. Evidence suggests MaR1’s benefits may extend to other systems. For example, MaR1 improves cardiac function and modulates macrophage switching in myocardial injury models [30]. It can induce physiological cardiac hypertrophy via the RORα/IGF‐1/PI3K/Akt pathway, relevant for diabetic cardiomyopathy [31]. MaR1 also shows vasculoprotective effects in atherosclerosis [32]. A MaR1 stereoisomer has been used to enhance mesenchymal stem cell therapy for diabetes and retinopathy [33]. Furthermore, MaR1 suppresses osteoblast ferroptosis and promotes ER‐phagy in diabetic osteoporosis [34, 35]. Thus, MaR1 may offer coordinated protection across key diabetic complication sites (kidney, heart, vasculature, and bone) by promoting inflammation resolution and immune homeostasis, as demonstrated by its M2‐polarizing effect in the kidney.

### 4.4. Rationale for Exogenous MaR1 Supplementation in DKD

The therapeutic potential of exogenous MaR1 is based on the concept of failed resolution in chronic inflammation. While direct measurements in DKD are evolving, patterns in chronic inflammatory diseases suggest SPMs production may become dysregulated or insufficient. For instance, serum MaR1 levels are reduced in DKD and postmenopausal psteoporosis patients [15, 36]. This creates a relative deficiency in resolution signals. Exogenous MaR1 administration aims to bypass this deficit, actively engaging proresolving pathways to steer the microenvironment from chronic damage toward repair, analogous to replacement therapy.

In this study, the successful reprograming of renal macrophages toward the proresolving M2 phenotype by exogenous MaR1 provides experimental validation for this therapeutic rationale, demonstrating that replenishing resolution signals can effectively break the vicious cycle of inflammation and tissue damage in DKD.

## 5. Limitations and Future Directions of the Study

This study has the following limitations: ① animal experiments were conducted only in a mouse model, and validation in other species such as rats or nonhuman primates is needed; ② cell model limitations: in vitro studies used BMDMs, which may not fully replicate the diversity of renal tissue‐resident macrophages. Future work should employ primary renal macrophages or relevant cell lines; ③ the optimal dosage and timing of MaR1 treatment were not determined, and further pharmacokinetic studies are required before clinical translation; and ④ our study did not include a dedicated mechanistic exploration of how MaR1 drives macrophage phenotypic switching. Future work should focus on elucidating the underlying signaling pathways and molecular mechanisms responsible for MaR1‐induced M2 polarization.

## 6. Conclusion

In summary, this study demonstrates that MaR1 exerts protective effects against DKD through multiple mechanisms. MaR1 significantly ameliorated metabolic disturbances, improved renal function, and alleviated interstitial fibrosis in diabetic mice. Both in vivo and in vitro, MaR1 promoted macrophage reprograming from the proinflammatory M1 phenotype toward the anti‐inflammatory M2 phenotype, accompanied by decreased production of IL‐6, TNF‐α, and iNOS and increased expression of IL‐10 and Arg‐1.

Collectively, our findings provide preclinical evidence that MaR1 improves DKD by integrating metabolic regulation with immune modulation. Although additional studies are required to validate causality and explore other potential signaling pathways, MaR1 represents a promising candidate for the development of targeted immunomodulatory therapies for DKD.

## Author Contributions

Xiumei Ma, Yueli Pu, and Yong Xu designed the study. Yueli Pu, Kang Geng, and Renliang Meng performed most of the experiments. Fangyuan Teng, Changying Zhao, and Yong Xu provided technical advice and fundings. Kang Geng, Yonglin Li, and Chunmei Zheng provided technical support for the main experiments. Xiumei Ma and Yueli Pu wrote the manuscript. Yong Xu, Changying Zhao, and Fangyuan Teng provided conceptual advice, edited and revised the manuscript.

## Funding

This work was supported by the Natural Science Foundation of China (Grants U22A20286 and 82470854), the Noncommunicable Chronic Diseases‐National Scienceand Technology Major Project (Grant 2024ZD0531300), the Sichuan Science and Technology Program (Grant 26ZRZZ0024), the Luzhou Science and Technology development grant (Grant 2024WGR205), and the Scientific research project of Southwest Medical University (Grant 2024LCYXZX02).

## Disclosure

All authors have read and approved the final manuscript.

## Conflicts of Interest

The authors declare no conflicts of interest.

## Data Availability

The datasets generated during and/or analyzed during the current study are available from the corresponding author upon reasonable request.
